# Proportion and determinants of adherence to antiretroviral therapy among HIV positive people registered under ART centre in South India

**DOI:** 10.1186/1471-2334-14-S3-P33

**Published:** 2014-05-27

**Authors:** V Hiregoudar, B Raghavendra, K Ramesh, ARB Sameena, K Hemagiri, S Basavaraj, T Gangadhara Goud, K Aravind, W Khan

**Affiliations:** 1 Vijayanagara Institute of Medical Sciences Bellary, Karnataka, India; 2 Mysore Medical College, Mysore, Karnataka, India

## Background

As on December 2012, there are about 6 lakh HIV positive people in India and around 83,000 HIV positive people in Karnataka are on ART. Among PLHIV, ART adherence is the second strongest predictor of progression to AIDS and death, after CD4 count. Consistently a very high level of adherence (> 95%) is required for ART to be effective on long term and to prevent the emergence of resistant viral strains and co-morbidities.

## Methods

A case series study was undertaken at ART centre, Bellary from June 2012 to May 2013. HIV positive subject aged above 15 years on ART for more than 6 months were included. Non probability purposive sampling was adopted. Data was collected by interviewing; treatment adherence was assessed by one week recall method.

## Results

A total of 536 HIV positive people were studied and out of which 67% [95%CI (62.89%- 70.83%)] of them reported >95% adherence to treatment. On univariate analysis factors like marital status, residential area, side effects to drugs, support of friends and family, knowledge regarding ART and tobacco usage had significant association with adherence (*p*<0.05). On logistic regression marital status (OR=1.591), area of residence (OR=1.939), side effects to drugs (OR=11.415), support of friends and family (OR=9.374), knowledge regarding ART (OR=2.344) and tobacco (OR=0.484) were independent factors influencing adherence.

## Conclusion

Socio demographic factors like marital status, residential area, personal factors like support of friends and family, adequate knowledge about ART and not using tobacco were found to show high level of ART adherence.

